# True Teachings

**Published:** 1874-03

**Authors:** 


### TRUE TEACHINGS.

Our sons are taught how to make money, and our daughters how to attract attention; but little if any thing is done toward imparting to them that instruction which would enable them to preserve and maintain unexceptionable health, without which, the admiration of courts is a bare endurance, and the glitter of costliest gems as valueless as the dust of the street.

